# Flow Cytometric Assessment of the Morphological and Physiological Changes of *Listeria monocytogenes* and *Escherichia coli* in Response to Natural Antimicrobial Exposure

**DOI:** 10.3389/fmicb.2018.02783

**Published:** 2018-11-14

**Authors:** Giacomo Braschi, Francesca Patrignani, Lorenzo Siroli, Rosalba Lanciotti, Oliver Schlueter, Antje Froehling

**Affiliations:** ^1^Campus Food Science, Department of Agricultural and Food Sciences, Alma Mater Studiorum, University of Bologna, Cesena, Italy; ^2^Leibniz Institute for Agricultural Engineering and Bioeconomy, Quality and Safety of Food and Feed, Potsdam, Germany

**Keywords:** food safety, non-thermal treatment, pathogens, stress response, membrane permeabilization

## Abstract

Essential oils (EOs) or their components represent one of the most promising natural, safe, and feasible alternatives to prevent the growth of food-borne pathogens like *Listeria monocytogenes* and *Escherichia coli* in food matrices. Although antimicrobial properties of EOs and their components are well-documented, limited and fragmented information is available on the changes induced by these compounds, even at sub-lethal concentrations, in the physiological properties of microbial cells. The aim of this study was to explore the morpho-physiological changes of *L. monocytogenes* Scott A and *E. coli* MG 1655 induced after 1 h exposure to different sub-lethal and lethal concentrations of citral, carvacrol, (E)-2-hexenal, and thyme EO. For this purpose, different cell viability parameters such as membrane integrity, esterase activity, and cytoplasmic cell membrane potential were measured by flow cytometry. Flow cytometric data revealed specific response patterns in relation to the strain, the natural antimicrobial and its concentrations. Both the target microbial strains showed an increased cell membrane permeabilization without a loss of esterase activity and cell membrane potential with increasing citral, carvacrol and thyme EO concentrations. By contrast, (E)-2-hexenal did not significantly affect the measured physiological properties of *L. monocytogenes* Scott A and *E. coli* MG 1655. The used approach allowed identifying the most effective natural antimicrobials in relation to the microbial target.

## Introduction

Consumer’s demand for minimally processed and ready-to-eat foods with a reduced content of synthetic preservatives has stimulated the research of alternative preservation strategies. Essential oils (EOs) or their components represent one of the most promising natural feasible alternatives to improve food safety, shelf-life and quality. Recognized as safe from international food authorities they are traditionally used in food industry as flavor and taste enhancers (Newberne et al., 2000). Their antimicrobial activity and the wide action spectra against several pathogenic and spoilage microorganisms are well-documented and several reviews are available (Burt, 2004; Hyldgaard et al., 2012; Tongnuanchan and Benjakul, 2014; Patel, 2015; Pandey et al., 2017). A wide literature documented their application as natural preservatives also in different food matrices, such as meat (Fratianni et al., 2010; Barbosa et al., 2015; Radha Krishnan et al., 2015), dairy products (Amatiste et al., 2014; Ehsani et al., 2016; Ben Jemaa et al., 2017), minimally processed fruits and vegetables (Patrignani et al., 2015; Siroli et al., 2015b,c) and beverages (Kiskó and Roller, 2005; Chueca et al., 2016). Among the natural antimicrobials, thyme EO, and some components of citrus and officinal EOs, such as citral, carvacrol, and (E)-2-hexenal, are very promising alternatives to traditional preservatives (Ivanovic et al., 2012). In fact, they are widely reported to be able to improve safety and shelf-life of several foods even when used at concentrations lower than their minimal bactericidal concentrations. In fact, their application in foods is subordinated to the usage of concentrations not detrimental for the product sensory properties (Zanini et al., 2014a,b; Silva-Angulo et al., 2015). In general, the cell wall, the cytoplasmic membrane and membrane proteins have been considered as the main targets of EOs and their components (Burt, 2004). However, the EO action mechanisms are reported to vary according to the natural antimicrobial and its concentration, the target species or strain, the food matrix, the storage conditions, etc. (Valero and Francés, 2006; Somolinos et al., 2008, 2010; Patrignani et al., 2013). As described by Burt (2004) and Hyldgaard et al. (2012), the primary site of toxic action of terpenes like citral and carvacrol is represented by the cell membrane due to their hydrophobic properties.

As reported by Somolinos et al. (2010), the exposure to different citral concentrations caused a disruption of the cytoplasmic cell membrane of *Escherichia coli* BJ4 and BJ4L1. A similar permeabilization effect was also observed for different *Listeria monocytogenes* strains. As reported by Zanini et al. (2014a,b), the exposure to citral and carvacrol increased the permeabilization of the cytoplasmic cell membrane and potentiated the activity of various antibiotics even at sub-lethal concentrations.

Additionally, the cytosol coagulation and the depletion of the microbial cell proton-motive force have been identified as action mechanisms of EOs (Burt, 2004; Hyldgaard et al., 2012; Patel, 2015). Aldehydes such as hexanal and (E)-2-hexenal are antimicrobials produced by plants and vegetable tissues which are damaged by biotic or abiotic stresses throughout the lipoxygenase pathway to prevent and/or inhibit the growth of plant pathogens (Lanciotti et al., 2004). It has been demonstrated that such tissue show a noticeable activity against several yeasts, molds, Gram-positive and Gram-negative bacterial strains of food interest (Nakamura and Hatanaka, 2002; Trombetta et al., 2002; Zhang et al., 2017) both in model and real food systems (Lanciotti et al., 1999, 2003; Siroli et al., 2014). As reported by Patrignani et al. (2008), (E)-2-hexenal acts as a surfactant and permeates by passive diffusion across the plasma membrane of many microorganisms. After reaching the cytoplasm, the α,β-unsaturated aldehyde is able to react with different nucleophilic groups (Kubo and Fujita, 2001; Lanciotti et al., 2004). Moreover, (E)-2-hexenal may cause cytoplasm coagulation as a result of thiol containing enzyme inhibition (Aiemsaard et al., 2011). The antimicrobial properties of thyme EO depend on its chemical composition and the target microorganism (Kim et al., 1995; Nevas et al., 2004; Sienkiewicz et al., 2011; Picone et al., 2013; Boskovic et al., 2015; Siroli et al., 2015a,c; Swamy et al., 2016).

Thyme EO is constituted of numerous different compounds, but its antimicrobial activity is mainly attributed to carvacrol and thymol. Thymol is structurally similar to carvacrol and they share their cellular targets. Studies have shown that thymol interacts with cell membrane permeability, leading to a depletion of membrane potential, cellular uptake of ethidium bromide, and leakage of potassium ions, ATP, and carboxyfluorescein (Helander et al., 1998; Lambert et al., 2001; Xu et al., 2008). Although literature regarding the action mechanisms of citral, carvacrol, (E)-2-hexenal, and thyme EO has been dramatically increased in the last years, the knowledge on their mechanisms on *L. monocytogenes* and *E. coli* is still fragmentary since it is affected by several factors such as concentration, strains, cell physiological state, treatment conditions, microbial interaction with exposure systems, etc.

In addition, antimicrobial activity of EOs and their components are not attributable to a specific mechanism but to the actions toward several cell targets. Moreover, for EOs a holistic approach should be considered, since synergistic actions among present components greatly affects their antimicrobial activities also at very low concentrations (Caccioni et al., 1997), and, consequently, the understanding of their action mechanisms becomes more complex. In addition, a heterogeneity in microbial population resistance to stress is reported to occur as a monomodal Gaussian with a narrow or broad distribution, or as a multimodal distribution comprising subpopulations of similar or vastly different numbers of individuals (Dhar and McKinney, 2007). However, the literature on the behavior of *L. monocytogenes* and *E. coli* cell populations exposed to natural antimicrobials is still scarce (Burt, 2004; Bakkali et al., 2008; Hyldgaard et al., 2012).

Flow cytometry represents a reliable and fast tool in food microbiology for the measurements of the changes on physiological single cell properties. By the use of the appropriate fluorescent dyes, it is possible to classify cells into three different categories: metabolically active, intact, or permeabilized cell mixtures (Hewitt and Nebe-Von-Caron, 2004; Johnson et al., 2013). Most common fluorescent dyes used in flow cytometry are fluorescent immune-conjugates and probes for fluorescence *in situ* hybridization and nucleic acid stains. In addition, several probes capable of measuring the membrane potential as well as cell enzymatic activity, viability, organelles, phagocytosis, development, and other properties are available (Haugland, 1994). Various authors demonstrated the suitability of flow cytometry to study the microbial cell responses even after the exposure to sub-lethal stress conditions (Luscher et al., 2004; Ananta et al., 2005; Berney et al., 2007; Mathys et al., 2007; Sunny-Roberts and Knorr, 2008; Da Silveira and Abee, 2009; Mols et al., 2010; Fröhling et al., 2012; Tamburini et al., 2013; Fröhling and Schlüter, 2015). In fact, this technique provides several information on the whole cell population and its changes during the exposure to stresses and the following recovery during storage. The knowledge of the behavior of the different sub-populations after exposure to natural antimicrobials is fundamental for their further application at industrial level as alternative to traditional preservatives, also to avoid resistance phenomena.

In this framework, the main aim was to investigate the potential of flow cytometry to study the changes of morphological and physiological properties of the food-borne pathogen *L. monocytogenes* Scott A and the indicator strain *E. coli* MG 1655, after 1 h exposure to different sub-lethal and lethal concentrations of citral, carvacrol, (E)-2-hexenal and thyme EO in order to clarify their specific action mechanisms and the responses of the whole cell population. For these purposes, different cell viability parameters, such as membrane integrity, esterase activity and cytoplasmic cell membrane potential were measured by flow cytometry.

## Materials and Methods

### Natural Antimicrobials

Some EOs (citral, carvacrol, and (E)-2-hexenal) used in these experiments were purchased from Sigma-Aldrich (Milan, Italy) while thyme EO was obtained from Flora s.r.l. (Pisa, Italy). Before conducting the experiments, EOs were properly diluted using absolute ethanol (Sigma-Aldrich, Milan, Italy) to prepare 100X EO stock solutions.

### Bacterial Strains

*Listeria monocytogenes* Scott A and *E. coli* MG 1655 were stored as glass bead cultures at -80°C for long-term preservation. To acclimatize cultures to the experimental conditions, one glass bead of each strain was given to 5 ml of Brain Heart Infusion broth (BHI) (Thermo-fisher, Milan, Italy) and incubated for 24 h without shaking at 37°C. After growth, cells were sub-cultured at 37°C for 24 h in BHI broth.

### Exposure to Natural Antimicrobials

In each assay, 250 mL of fresh BHI broth was inoculated with 2.5 mL of bacteria suspension (corresponding to 1% of the final volume) to reach a 4 log CFU/mL concentration and incubated without stirring at 37°C. The growth was monitored by measuring the optical density (OD) at λ = 600 nm. For *L. monocytogenes* Scott A the exposure was performed in the middle of the exponential growth phase while for *E. coli* MG 1655 the exposure was performed in the stationary growth phase (OD = 2, λ = 600 nm). For both microbial strains, 200 μL of natural antimicrobial hydro alcoholic stock solutions was added to 20 mL of liquid cultures in order to obtain the concentrations reported in Tables 1, 2. Cultures were incubated for 1 h at 37°C.

**Table 1:** Essential oils, their components, and relative concentrations, used for the treatments of *L. monocytogenes Scott A.*

Natural antimicrobial	Concentration tested (mg/L)
Citral	50^1^, 85, 125, 250 mg/L^1^
Carvacrol	20^1^, 35, 50, 100 mg/L^1^
(E)-2-hexenal	150^1^, 250, 400, 800 mg/L^1^
Thyme essential oil	40^1^, 70, 100, 200 mg/L^1^


*^*1*^MIC value tested for *Listeria monocytogenes* Scott A.*

**Table 2:** Essential oils, their components, and relative concentrations, used for the treatments of *Escherichia coli* MG 1655.

Natural antimicrobial	Concentration tested (mg/L)
Citral	200, 330, 500, 1000^1^, 3000 mg/L^2^
Carvacrol	25, 40, 60, 120^1^, 250 mg/L^2^
(E)-2-hexenal	80, 135, 200, 400^1^, 425 mg/L^2^
Thyme essential oil	50, 86, 125, 250^1^, 300 mg/L^2^


*^*1*^MIC value tested for *Escherichia coli* MG 1655; ^2^MBC value tested for *Escherichia coli* MG 1655.*

For *L. monocytogenes* Scott A the natural antimicrobial operative concentrations were determined according to the minimum inhibitory concentration standard method (MIC) as previously described by Siroli et al. (2015a,b). For each selected natural antimicrobial, *L. monocytogenes* cells were exposed to three sub-lethal concentrations corresponding to the 1/5, 1/3, 1/2 of the MIC value as well as to the MIC values (Table 1). For *E. coli* MG 1655, the minimum inhibitory concentration (MIC) and minimum bactericidal concentration (MBC) of the selected natural antimicrobials were determined in preliminary studies according to the protocol described by Siroli et al. (2015a,b) (data not shown). *E. coli* MG 1655 cells were exposed to different natural antimicrobials in sub-lethal concentrations (corresponding to 1/5, 1/3, 1/2 of the MIC values) as well as to the MIC and MBC values (Table 2). After exposure, the total viable cell count of *L. monocytogenes* Scott A and *E. coli* MG 1655 was immediately performed. Afterward, bacterial cells were harvested by centrifugation at 3214 × *g* and 4°C for 15 min, resuspended in 250 μL of phosphate buffered saline (PBS, 50 mM) and centrifuged at 7000 × *g* and 4°C for 5 min. For the subsequent staining procedures and flow cytometric analysis, *L. monocytogenes* pellets were resuspended in 100 μL (50 mM) PBS, while *E. coli* samples were resuspended in 100 μL (50 mM) Tris buffer. For both microorganisms the final cell density of each sample was about 10^9^ cells/mL.

### Total Viable Cell Count

The total viable cell count of bacteria after exposure to EOs or their components was determined by plate count methods in duplicate. Samples were serially diluted in microtest plates (96er U-profile, Carl Roth GmbH & Co KG, Germany) using physiological saline buffer (9 g/L NaCl) as dilution solution. 100 μL of each dilution was spread on BHI agar (Thermo Fisher Scientific, Ltd., Milan, Italy) and the growth (colony forming units) was evaluated after 24 h at 37°C.

### Flow Cytometric Analysis

All experiments were performed using a CyFlow ML flow cytometer (Sysmex Partec GmbH, Görlitz, Germany) equipped, among others, with a 50 mW blue solid state laser emitting at a wavelength of 488 nm. A photomultiplier with a band pass filter of 536 ± 20 nm was used to collect fluorescence data of thiazole orange (TO), carboxyfluorescein (cF), and green DiOC_2_(3), while the fluorescence of propidium iodide and red DiOC_2_(3) was recorded in the photomultiplier with a band pass filter of 620 ± 11 nm. To correct the overlap of one dye’s emission into another dye’s detector, fluorescence signal compensation was performed. Data obtained from each photomultiplier channels were collected as logarithmic signals and analyzed using the FloMax software 3.0 (Sysmex Partec GmbH, Görlitz, Germany). For each sample, one hundred thousand events were measured at a flow rate of approximately 3000 events/sec. According to Fröhling et al. (2012), the density plots obtained by flow cytometric analyses were divided into four regions. Each region is associated with cells revealing different physiological or morphological properties. The average of the percentage values obtained from three density plots was calculated and illustrated as diagrams where the treatment concentration and the percentage of fluorescent cells is displayed by the x-axis and the y-axis, respectively. The different cell parameters investigated during this experimentation were: cell membrane integrity (TO-PI staining), cell membrane potential (DiOC_2_(3) staining), and cell membrane integrity and esterase activity (cF-PI staining) as indicator of the microbial population viability. Staining procedures were performed as described earlier by Fröhling and Schlüter (2015) with some adaptations to the bacterial species used.

#### Membrane Integrity

Cell membrane integrity was evaluated after the treatments using a combination of thiazole orange (TO) and propidium iodide (PI) dyes (Sigma-Aldrich KGaA, Darmstadt, Germany). Staining procedures were performed in the dark with some variations depending on the microorganisms tested.

For *L. monocytogenes*, 20 μL of resuspended pellets was diluted in PBS (50 mM) to a cell density of approximately 10^6^ cells/mL. Thiazole orange (0.2 μM) was added to the samples and incubated for 10 min at room temperature. After incubation, 30 μM propidium iodide was added and samples were analyzed after 5 min.

As described above, *E. coli* resuspended pellets were diluted in Tris buffer (50 mM) to a cell density of approximately 10^6^ cells/mL. Thiazole orange was added to a final concentration of 2.5 μM and samples were incubated for 15 min at room temperature. Propidium iodide staining was performed as described for *L. monocytogenes* samples.

#### Esterase Activity and Membrane Permeabilization

The cell esterase activity and the membrane integrity were evaluated after the exposure to natural antimicrobials using 5(6)-carboxyfluorescein diacetate mixed isomers (cFDA) (Sigma-Aldrich, KGaA, Darmstadt, Germany) and PI. A volume of 60 μL of concentrated *L. monocytogenes* samples were stained with an equal volume of cFDA (200 μM) stock solution to obtain a final cFDA concentration of 100 μM, incubated at 37°C in a water bath for 5 min and centrifuged at 7000 × *g* and 4°C for 5 min. Cell pellets were diluted 1:1 in PBS (50 mM). The PI staining procedure was performed as previously described. The cFDA staining procedure for *E. coli* followed the protocol described for *L. monocytogenes* with some differences. Cells were incubated with 833 μM cFDA (in 50 mM Tris) at 37°C in a water bath for 45 min and then centrifuged at 7000 × *g* and 4°C for 5 min. The pellets were resuspended in 60 μL Tris (50 mM) and stained with 30 μM PI. Samples were analyzed after 10 min of incubation.

#### Membrane Potential

The membrane potential of bacteria cells was measured using 3,3′-diethyloxacarbocyanine iodide [DiOC_2_(3)] provided by Sigma-Aldrich, Germany. The protocol applied was in agreement with Novo et al. (1999) and Fröhling and Schlüter (2015) with further modifications. *L. monocytogenes* suspensions were diluted in 50 mM PBS containing 10 mM D-Glucose and 30 μM DiOC_2_(3) and then incubated for 15 min at room temperature in the dark. Afterward the suspension was centrifuged at 7000 × *g* and 4°C for 5 min and the pellet was resuspended in 1 mL PBS (50 mM). *E. coli* staining procedure required a different staining buffer due to the higher complexity of the outer and inner cell membrane. First, samples were suspended in 10 mM D-Glucose, 30 μM DiOC_2_(3) and EDTA (0.5 mM) Tris buffer (50 mM). After the centrifugation and the incubation time as previously described, *E. coli* samples were resuspended in 1 mL of Tris (50 mM). After the staining procedure samples were immediately analyzed to detect shifts in the cell membrane potential. The ratio of the mean red to the mean green DiOC_2_(3)-fluorescence channel value was calculated to investigate changes in the membrane potential. Due to the chosen cytometer settings the red/green DiOC_2_(3)-fluorescence ratio of depolarized cells was ≤1. It was assumed that the red/green ratio of untreated cells represents the relative membrane potential of intact cells (Novo et al., 1999). A reduction of the red/green ratio reflects the loss of cell membrane potential.

### Statistical Analysis

Statistical analyses were performed to evaluate significant differences between samples using the R software (R Core Development Team, 2017). One way-ANOVA with Tukey test with a significance level of 0.05 was used.

## Results

### Treatment Effects on Total Viable Count of *Listeria monocytogenes* Scott A

The exposure of *L. monocytogenes* cells to the natural antimicrobials at different concentrations was performed in the middle of the exponential growth phase (OD = 0.4; λ = 600 nm). In all trials, the cell loads before the treatments were about 8.8 log CFU/mL (Tables 3, 4). After 1 h exposure, the untreated controls and the samples exposed to 1% ethanol showed the same cell counts. Only the 1 h exposure to carvacrol and thyme EO resulted in a significant reduction of the total viable counts at the highest concentration tested (Tables 3, 4). More specifically, the exposure to 100 mg/L of carvacrol which is corresponding to the MIC value, reduced the viable cell load by one logarithmic cycle (7.59 log CFU/mL) (Table 3B). A more severe effect was observed after the exposure to thyme EO, reducing the viable counts to 7.45 log CFU/mL and 5.23 CFU/mL after the exposure to 100 and 200 mg/L, respectively (Table 4D). In contrast, 1 h exposure to citral and (E)-2-hexenal produced no significant effect on cell loads of *L. monocytogenes* Scott A cells (Tables 3A, 4C).

**Table 3:** Total viable counts of *Listeria monocytogenes* Scott A after 1 h exposure to different concentrations of Citral **(A)** and Carvacrol **(B)**.

A	log CFU/mL		*SD*	B	log CFU/mL		*SD*
Untreated control	8.79	±	0.10^a^	Untreated control	8.81	±	0.17^a^
EtOH 1%	8.95	±	0.23^a^	EtOH 1%	8.7	±	0.18^a^
Citral 50 mg/L	8.86	±	0.11^a^	Carvacrol 20 mg/L	8.75	±	0.23^a^
Citral 85 mg/L	8.59	±	0.10^a^	Carvacrol 35 mg/L	8.79	±	0.09^a^
Citral 125 mg/L	8.88	±	0.14^a^	Carvacrol 50 mg/L	8.79	±	0.16^a^
Citral 250 mg/L (MIC)	8.69	±	0.08^a^	Carvacrol 100 mg/L (MIC)	7.59	±	0.14^b^


*Different letters indicate that data are significantly different (*p* < 0.05).*

**Table 4:** Total viable counts of *Listeria monocytogenes* Scott A after 1 h exposure to different concentrations of (E)-2-hexenal **(C)** and Thyme EO **(D)**.

C	log CFU/mL		*SD*	D	log CFU/mL		*SD*
Untreated control	8.87	±	0.22^a^	Untreated control	8.71	±	0.17^a^
EtOH 1%	8.99	±	0.10^a^	EtOH 1%	8.59	±	0.14^a^
(E)-2-Hexenal 150 mg/L	8.99	±	0.23^a^	Thyme EO 40 mg/L	8.72	±	0.07^a^
(E)-2-Hexenal 250 mg/L	9.13	±	0.11^a^	Thyme EO 70 mg/L	8.69	±	0.28^a^
(E)-2-Hexenal 400 mg/L	9.00	±	0.09^a^	Thyme EO 100 mg/L	7.45	±	0.72^b^
(E)-2-Hexenal 800 mg/L (MIC)	8.49	±	0.10^a^	Thyme EO 200 mg/L (MIC)	5.23	±	0.15^c^


*Different letters indicate that data are significantly different (*p* < 0.05).*

### Treatment Effects on Total Viable Count of *Escherichia coli* MG 1655

*Escherichia coli* MG 1655 samples were exposed to EO or their bioactive compounds at the beginning of the stationary growth phase (OD = 2; λ = 600 nm). The cell loads of *E. coli* before the exposure to the natural antimicrobials ranged between 8.5 and 9.0 log CFU/mL (Tables 5, 6). Analogously to *L. monocytogenes*, no differences on the cell loads were highlighted between the untreated controls and the samples exposed to 1% EtOH (Tables 5, 6). The highest effect on *E. coli* was observed after the exposure to citral, even at lowest concentration tested. The exposure to 200 mg/L reduced the viable cell load to 7.22 log CFU/mL. Increasing concentrations reduced the total viable counts to values ranging between 6.60 and 6.12 CFU/mL. The exposure to the citral MBC concentration caused a reduction of cell loads below the detection limit (Table 5A). Also thyme EO and carvacrol treatments significantly decreased the total viable cell loads. A reduction of three logarithmic cycles were observed after the exposure to carvacrol MBC concentration (250 mg/L), while an exposure to thyme EO MIC and MBC concentrations reduced cell load to 6.52 and 5.59 log CFU/mL, respectively (Tables 5B, 6D). (E)-2-hexenal exposure did not affect the cell loads of *E. coli* (Table 6C).

**Table 5:** Total viable count of *Escherichia coli* MG 1655 after 1 h exposure to Citral **(A)** and Carvacrol **(B)**.

A	log CFU/mL		*SD*		B	log CFU/mL		*SD*
Untreated control	8.97	±	0.24^a^		Untreated control	8.9	±	0.16^a^
EtOH 1%	8.96	±	0.15^a^		EtOH 1%	8.98	±	0.21^a^
Citral 200 mg/L	7.22	±	0.20^b^		Carvacrol 25 mg/L	8.65	±	0.09^a^
Citral 330 mg/L	6.6	±	0.13^c^		Carvacrol 40 mg/L	9.02	±	0.11^a^
Citral 500 mg/L	6.28	±	0.18^c^		Carvacrol 60 mg/L	8.75	±	0.25^a^
Citral 1000 mg/L (MIC)	6.12	±	0.20^d^		Carvacrol 125 mg/L (MIC)	8.79	±	0.22^b^
Citral 3000 mg/L (MBC)	-^∗^				Carvacrol 250 (MBC)	4.85	±	0.47^c^


*^∗^Detection limit 1 log CFU/mL.*

*Different letters indicate that data are significantly different (*p* < 0.05).*

**Table 6:** Total viable count of Escherichia *coli* MG 1655 after 1 h exposure to (E)-2-hexenal **(C)** and Thyme EO **(D)**.

C	log CFU/mL		*SD*		D	log CFU/mL		*SD*
Untreated control	8.88	±	0.26^a^		Untreated control	8.76	±	0.24^a^
EtOH 1%	9	±	0.23^a^		EtOH 1%	8.63	±	0.22^a^
(E)-2-Hex. 80 mg/L	8.93	±	0.20^a^		Thyme EO 50 mg/L	8.53	±	0.32^a^
(E)-2-Hex. 135 mg/L	8.78	±	0.20^a^		Thyme EO 85 mg/L	8.63	±	0.35^a^
(E)-2-Hex. 200 mg/L	8.89	±	0.22^a^		Thyme EO 125 mg/L	8.86	±	0.53^a^
(E)-2-Hex. 400 mg/L (MIC)	8.83	±	0.09^a^		Thyme EO 250 mg/L (MIC)	6.52	±	0.04^c^
(E)-2-Hex. 425 mg/L (MBC)	8.64	±	0.23^a^		Thyme EO 300 mg/L (MBC)	5.59	±	0.26^d^


*Different letters indicate that data are significantly different (*p* < 0.05).*

### Treatment Effects on the Membrane Integrity of *Listeria monocytogenes* Scott A

**FIGURE 1:**
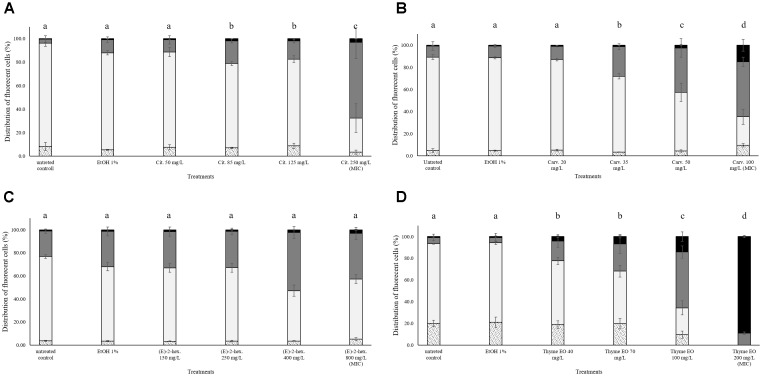
Membrane integrity of *Listeria monocytogenes* Scott A after 1 h exposure to different concentrations of natural antimicrobials: Citral **(A)**, Carvacrol **(B)**, (E)-(2)-hexenal **(C)**, and thyme EO **(D)**. White bars with dots represent cell fragments or unstained cells; White bars represent cells with intact cell membrane; Gray bars represent cells with slight cell membrane permeabilization; Black bars represent cells with (complete) cell membrane permeabilization. Different letters indicate that data are significantly different (*p* < 0.05).

The exposure of *L. monocytogenes* Scott A to different natural antimicrobials caused an augment of the fractions with cells having slightly permeabilized cell membranes (PI + TO stained cells) and permeabilized cell membranes (PI stained cells). The distribution of stained cells varied according to different treatments and concentrations used. The percentage of *L. monocytogenes* Scott A with intact cell membranes remained almost constant (above 80%) after exposure to 1% ethanol in all the performed trials (Figure 1). The different concentrations of citral (Figure 1A) increased the percentages of cells with slightly permeabilized cell membranes. The magnitude of the damaging effect rose with increasing citral concentrations. In fact, at the highest concentration (250 mg/L) 60% of stained cells showed a slightly permeabilized cell membrane. A value of about 3% cells with permeabilized cell membrane was observed in all the conditions independently on the severity of chemical stress applied (citral concentration). A similar pattern was observed for carvacrol (Figure 1B). All concentrations tested induced a significant reduction in the population with intact cell membranes. In particular, while in the control samples the population with intact cell membranes was about 80%, this value decreased to 25% in the samples exposed to 100 mg/L carvacrol (MIC value). Simultaneously, the percentage of cells with slightly permeabilized cell membranes increased from 12 to 50% augmenting the carvacrol concentrations. The exposure to the MIC carvacrol concentration also raised the percentage of cells with permeabilized cell membranes up to 15%. Compared to the untreated control, the exposure to (E)-2-hexenal had no significant effect on the cell membrane integrity of *L. monocytogenes* Scott A (Figure 1C). Thyme EO had the highest effect on the cell membrane integrity compared to the other natural antimicrobials tested. The percentage of slightly permeabilized and completely permeabilized cells increased with the treatment concentrations, and the exposure to 200 mg/L of thyme EO (MIC concentration) induced a complete membrane permeabilization in more than 90% of the cell population (Figure 1D). Except for Thyme EO exposure, the percentage values of unstained cells/cell fragments were lower than 3% independently on the antimicrobial and its concentration (Figure 1).

### Treatment Effects on the Esterase Activity and Membrane Integrity of *Listeria monocytogenes* Scott A

**FIGURE 2:**
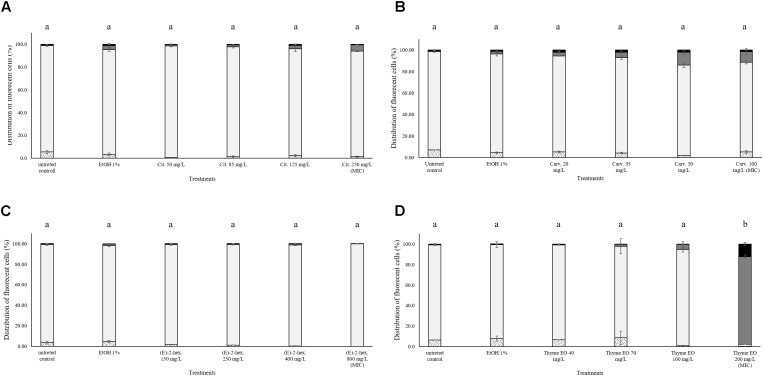
Esterase activity and membrane integrity of *L. monocytogenes* Scott A after 1 h exposure to natural antimicrobials: Citral **(A)**, Carvacrol **(B)**, (E)-(2)-hexenal **(C)**, and thyme EO **(D)**. White bars with dots represent cell fragments or unstained cells; White bars represent cells with intact cell membrane and esterase activity; Gray bars represent cell membrane permeabilization but still existing esterase activity; Black bars represent cells with cell membrane permeabilization but without esterase activity. Different letters indicate that data are significantly different (*p* < 0.05).

No effects on the esterase activity of *L. monocytogenes* cells were evidenced after the exposure to natural antimicrobials or their bioactive compounds. As shown in Figure 2, the percentage of fluorescent cells with intact cell membranes and esterase activity was constantly above 80% with the only one exception of thyme EO 200 mg/L exposure. The MIC thyme EO exposure caused a complete permeabilization of the cells, however, these cells still showed esterase activity (Figure 2D).

### Treatment Effects on the Cell Membrane Potential of *Listeria monocytogenes* Scott A

**FIGURE 3:**
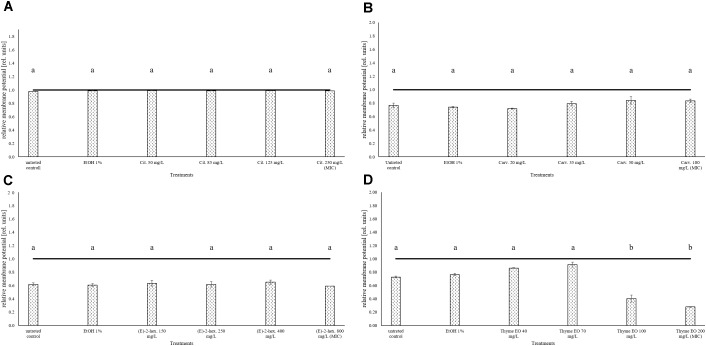
Relative membrane potential of *L. monocytogenes* Scott A, expressed as red/green ratio of DiOC_2_(3)-fluorescence intensity, after 1 h exposure to natural antimicrobials: Citral **(A)**, Carvacrol **(B)**, (E)-(2)-hexenal **(C)**, and thyme EO **(D)**. The black line represents the threshold between polarized (ratio > 1) and depolarized (ratio < 1) cells. Different letters indicate that data are significantly different (*p* < 0.05).

The measurement of the relative membrane potential using 3,3′-Diethyloxacarbocyanine iodide DiOC_2_(3) showed no significant differences between untreated and treated cells of *L. monocytogenes* Scott A. In fact, red/green ratios were always lower than the value 1 independently on the treatments and concentrations used (Figure 3).

### Treatment Effects on Membrane Integrity of *Escherichia coli* MG 1655

**FIGURE 4:**
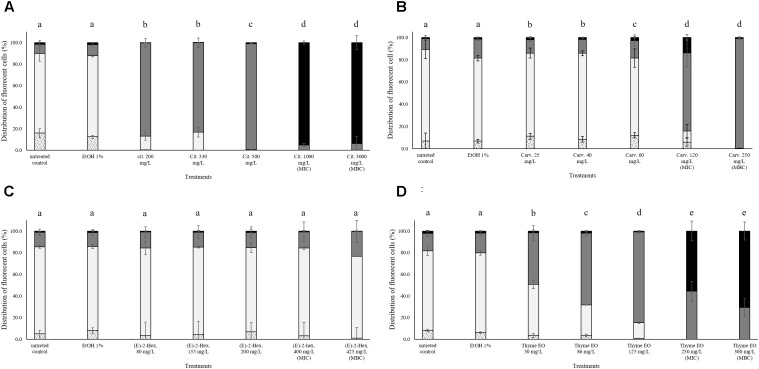
Membrane integrity of *Escherichia coli* MG 1655 after 1 h exposure to different concentrations of natural antimicrobials: Citral **(A)**, Carvacrol **(B)**, (E)-(2)-hexenal **(C)**, and thyme EO **(D)**. White bars with dots represent cell fragments or unstained cells; White bars represent cells with intact cell membranes; Gray bars represent cells with slightly permeabilized cell membranes; Black bars represent cells with (complete) with permeabilized cell membranes. Different letters indicate that data are significantly different (*p* < 0.05).

In all trials, the percentage of *E. coli* stained only with TO (cells with intact cell membrane) remained almost constant (above 80%) after the exposure to 1% ethanol (Figure 4). Citral determined a significant effect on cell membrane integrity even at sub-lethal concentrations (Figure 4A). After exposure to 200–300 mg/L citral, the percentages of cells with slightly permeabilized cell membranes were higher than 80%. No cells with intact cell membrane were found after the exposure to citral MIC and MBC values (Figure 4A). Minor impacts on *E. coli* cell membrane integrity were evidenced after the exposure to carvacrol and (E)-2-hexenal, independently on their sub-lethal concentrations (Figures 4B,C). Only the exposure to carvacrol inhibition and bactericidal concentrations (120–250 mg/L) triggered cell membrane permeabilization (Figure 4B). The effect of thyme EO on the cell membrane was dependent on the applied concentration. The fluorescence signal of intact cells decreased (80–20%) with an increase of the treatment concentration, while the percentage of cells with permeabilized cell membranes increased simultaneously (Figure 4D).

### Treatment Effects on Esterase Activity and Membrane Integrity of *Escherichia* coli MG 1655

**FIGURE 5:**
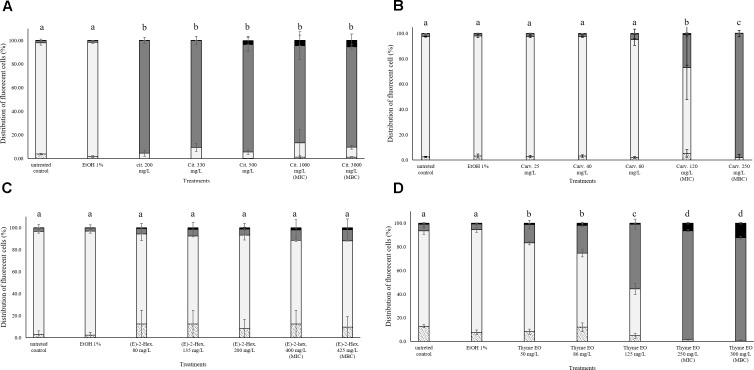
Esterase activity and membrane integrity of *E. coli* MG 1655 after 1 h exposure to natural antimicrobials: Citral **(A)**, Carvacrol **(B)**, (E)-(2)-hexenal **(C)**, and thyme EO **(D)**. White bars with dots represent cell fragments or unstained cells; White bars represent cells with intact cell membrane and esterase activity; Gray bars represent cell membrane permeabilization but still existing esterase activity; Black bars represent cells with cell membrane permeabilization but without esterase activity. Different letters indicate that data are significantly different (*p* < 0.05).

The exposure to citral induced cell membrane permeabilization of *E. coli* without a loss of esterase activity. The percentage of fluorescent cells with permeabilized cell membranes but still existing esterase activity was higher than 80% independent of the natural antimicrobial concentrations. Only the exposure to bactericidal concentrations (1000 and 3000 mg/L citral) increased (10%) the amounts of the populations with permeabilized cell membranes and without esterase activity (Figure 5A). Minor impacts on *E. coli* cell membrane integrity and esterase activity were evidenced after the exposure to carvacrol and (E)-2-hexenal. *E. coli* samples treated with the bactericidal concentrations (120 and 250 mg/L) of carvacrol showed a cell membrane permeabilization without a loss of esterase activity (Figure 5B). The exposure to (E)-2-hexenal increased the percentage of cell fragments or unstained cells, independently on the concentration used. They represented about the 15% of the whole population in all the conditions tested (Figure 5C). The effects of thyme EO on the cell membrane and esterase activity were related to the concentration tested. The fluorescence signal of the intact cells decreased (80–30%) with the exposure to the sub-lethal concentration tested while the percentage of cell populations with permeabilized cell membranes and esterase activity increased (Figure 5D). A significant loss in the cell esterase activity was only observed after the exposure to 250 and 300 mg/L thyme EO.

### Treatment Effects on Cell Membrane Potential of *Escherichia coli* MG 1655

**FIGURE 6:**
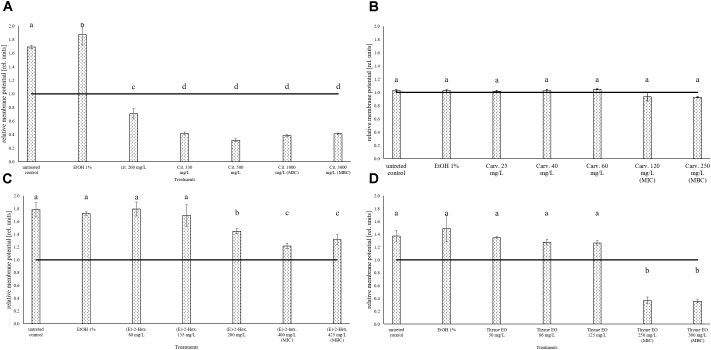
Relative membrane potential of *E. coli* MG 1655, expressed as red/green ratio of DiOC_2_(3)-fluorescence intensity, after 1 h exposure to natural antimicrobials: Citral **(A)**, Carvacrol **(B)**, (E)-(2)-hexenal **(C)**, and thyme EO **(D)**. The black line represent the threshold between polarized (ratio > 1) and depolarized (ratio < 1) cells. Different letters indicate that data are significantly different (*p* < 0.05).

The measurement of the relative membrane potential using DiOC_2_(3) showed that the untreated *E. coli* cells had a red/green ratio of 1.69 before the exposure to citral (Figure 6A). The value was reduced below 1 independently of the concentration used, suggesting the capability of citral to depolarize the cell membrane of *E. coli*. A membrane depolarization was also observed for the MIC and MBC values of thyme EO (Figure 6D). No cell membrane depolarization was observed after the exposure to carvacrol and (E)-2-hexenal (Figures 6B,C) but (E)-2-hexenal induced a concentration dependent reduction of the cell membrane potential.

## Discussion

As described by many authors, EOs and their bioactive compounds are characterized by an antimicrobial activity both *in vitro* and in real food systems. Although their antimicrobial properties are well-documented, only limited information is available about their mechanisms of action on *E. coli* and *L. monocytogenes* (Lambert et al., 2001; Burt, 2004; Bakkali et al., 2008; Xu et al., 2008; Hyldgaard et al., 2012; Picone et al., 2013). Additionally, the literature on the responses of the whole cell populations of the selected species to EOs is still scarce (Xu et al., 2008). However, cell wall, membrane and energetic pathways are generally considered as the main microbial cell targets of EOs (Burt, 2004; Bakkali et al., 2008; Hyldgaard et al., 2012). In this framework multiparametric flow cytometric analyses were performed in order to assess the effects of citral, carvacrol, (E)-2-hexenal and thyme EO on the whole microbial populations of the two target microorganisms. Consequently, their effects on membrane integrity, esterase activity and cell membrane potential were investigated. Flow cytometric data of *L. monocytogenes* Scott A and *E. coli* MG 1655 populations after 1 h stress exposure revealed specific response patterns in relation to the natural antimicrobials and their concentrations. Concerning the membrane permeabilization, the percentage of permeabilized cells raised with the antimicrobial concentration applied, with the only exception for (E)-2-hexenal. The membrane integrity was analyzed using dye exclusion methods with thiazole orange (TO) and the divalent propidium iodide (PI) as dyes. In fact, TO is able to pass thought lipidic bilayers and to stain both DNA or RNA while PI, due to multiple charges, can only react with nucleic acids when membrane is disrupted or permeabilized (Kim et al., 2009; Díaz et al., 2010). Both target strains showed an increased cell membrane permeabilization with an increase of citral, carvacrol, and thyme EO concentrations. However, *E. coli* reacted sensitively to all the natural antimicrobials. In fact, they induced more severe membrane permeabilization and cell load reductions of *E. coli* in comparison to *L. monocytogenes*. The most effective antimicrobial against *E. coli* was citral. Actually, *E. coli* showed the highest cell load reduction and membrane permeabilization after the exposure to citral. A wide literature shows that the outer membrane of Gram-negative bacteria, which acts as a barrier against macromolecules and hydrophobic substances, increase their resistance to several antimicrobials including many EO (Nikaido and Vaara, 1985; Helander et al., 1997). However, also Somolinos et al. (2008) demonstrated that citral was more effective on *E. coli* J1 than on *L. monocytogenes* NCTC11994 under different experimental conditions, and especially at pH 7. These authors, using fluorescence microscopy and propidium iodide as dye, demonstrated that citral disrupted the outer cell envelope of *E. coli*, forming pores and permitting the cytoplasm entrance of molecules with a size of 660 Da. To destabilize the lipopolysaccharide layer of the outer membrane, the use of several chelating agents, such as EDTA, citric acid, and other substances, as well as high pressure homogenization have been proposed (Cutter and Siragusa, 1995; Helander et al., 1997; Vannini et al., 2004; Patrignani et al., 2010).

Different authors reported that citral and other low molecular mass ketons are sufficiently hydrophilic to pass throughout porin proteins to the deeper parts of Gram-negative bacteria without destabilization of the outer membrane (Helander et al., 1997; Lanciotti et al., 2003; Belletti et al., 2004; Belletti et al., 2008).

Carvacrol and thyme EO significantly reduced *E. coli* cell loads but only when used at MIC and MBC values. Only these concentrations induced a significant membrane permeabilization of *E. coli* cells. These data are in agreement with those of Xu et al. (2008) obtained by flow cytometry after exposure of *E. coli* AS1 90 to 200 mg/L of carvacrol and thymol, the major constituent of thyme EO. These authors showed cell membrane permeabilization processes associated with significant reductions of the cell loads (Xu et al., 2008). Also Gill and Holley (2006), using confocal laser scanning microscopy, showed a clear membrane disruption of *E. coli* O157:H7 after 10 min exposure to eugenol and carvacrol used at concentrations able to reduce the pathogen viability of about 8 log cycles.

A concentration dependent permeabilization process was also evidenced for *L. monocytogenes* Scott A after exposure to citral, carvacrol, and thyme EO. No literature is available on the effects of such antimicrobials on *L. monocytogenes* membrane permeabilization. However, Ultee et al. (1999) showed for Gram-positive *Bacillus cereus* that carvacrol caused an increased membrane permeability to cations such as H^+^ and K^+^. Also, no significant *L. monocytogenes* cell load reductions were highlighted for citral, independent of the concentration used. Somolinos et al. (2008) also showed a scarce sensitiveness of *L. monocytogenes* ATCC19114 serotype 4a to citral. Compared to citral, carvacrol and thyme EO were more effective on *L. monocytogenes*. However, significant cell load reductions were only observed using the MIC value of carvacrol and thyme EO concentrations. Friedman et al. (2002) described how carvacrol and thymol had the highest effect on the cell loads of *L. monocytogenes* RM2199 and RM2388 compared to other 23 EO constituents using a microplate assay. The effects of the natural antimicrobials on the esterase activity of *L. monocytogenes* and *E. coli* was measured because it is considered as reliable way to evaluate the cell damages induced after several antimicrobial treatments (Díaz et al., 2010; Surowsky et al., 2014; Fröhling and Schlüter, 2015; Hong et al., 2015; Combarros et al., 2016; Meng et al., 2016). In fact, carboxyfluorescein diacetate (cFDA), the fluorescent dye used to measure esterase activity, is a lipophilic non-fluorescent compound which is hydrolyzed in the cytoplasm to the fluorescent carboxyfluorescein (cF) by unspecific esterases. According to several literature data only cells with integer membrane and active intracellular enzymes remain fluorescent (Haugland, 1999; Hoefel et al., 2003; Fröhling and Schlüter, 2015; Reineke et al., 2015). However, the data obtained in our experimental conditions showed for both target strains that this enzymatic activity was not affected by the exposure to the antimicrobials used, independently on their concentrations. This data seems in disagreement with literature showing that membrane permeabilization is generally associated to the losses of esterase activity (Hayouni et al., 2008; Paparella et al., 2008; Xu et al., 2008). A decrease of esterase activity which was associated to membrane permeabilization was observed also in *L. monocytogenes* and *E. coli* exposed to EOs or their components (Xu et al., 2008). However the literature data evidenced losses of esterase activity only when lethal antimicrobial concentrations were used. For example, Paparella et al. (2008) showed by flow cytometric analyses that *L. monocytogenes* ATCC19114 serotype 4a had significant reductions of both esterase activity and cell viability after 1 h exposure to emulsified cinnamon, oregano, or thyme EOs (Paparella et al., 2008). However, these Authors tested the effects of emulsions having EO concentrations ranging between 0.02 and 0.5%. These concentrations are generally lethal concentrations for *L. monocytogenes* independently on the exposure conditions. However, they are not compatible with any usage in food systems due to the low sensory thresholds of the natural antimicrobials tested. In our experimental conditions, probably due to the use of concentrations significantly lower to those tested in the literature, the amount of cFDA hydrolyzed into cF after the exposure to natural antimicrobials remained constant, independently on the cell membrane permeabilization degree and the microorganism considered. In addition, the membrane potential of *L. monocytogenes* was not affected by the 1 h exposure to the natural antimicrobials considered. The membrane potential of cells is considered as fundamental in numerous processes of the live cell physiology and it is strongly related to bacterial viability (Novo et al., 1999). In fact, according to the literature, only living cells are able to maintain membrane potential (Díaz et al., 2010). In our experimental conditions, also at MIC values, the antimicrobials tested did not affect the membrane potential of *L. monocytogenes* compared to the control ones. However, also at MIC values of the tested antimicrobials, *L. monocytogenes* cells showed only a slight presence or even a complete absence of permeabilization of the cell membrane. By contrast, *E. coli* compared to *L. monocytogenes*, showed with a higher sensitiveness to almost all the antimicrobials considered, showed also cell membrane depolarization depending on the antimicrobial used and its concentration. A complete depolarization (red/green ratio below 1) of *E. coli* cells was observed for all citral concentrations tested and thyme EO MIC and MBC values. Also Kim and Kang (2017) observed that the cell membrane potential of *E. coli* O157:H7 was significantly reduced after exposure to citral and thymol combined with an ohmic heating treatment, both in model and real food systems.

In our experimental conditions, also (E)-2-hexenal exposure caused a reduction of relative membrane potential of the *E. coli* population. In contrast, no effect on the membrane potential of *E. coli* was evidenced after carvacrol treatments. Different studies confirmed that Gram-negative bacteria, due the outer membranes, were characterized by a higher resistance to carvacrol (Kokoska et al., 2002; Okoh et al., 2010). The reduction of membrane potential after antibacterial treatments is considered fundamental for pathogenic species, since viable but not culturable cells are still able to cause diseases (Fröhling and Schlüter, 2015).

In general, the data obtained indicated that sub-lethal treatments had minor impacts on *L. monocytogenes* compared to *E. coli*. In fact, all the antimicrobial tested induced only a slight cell membrane permeabilization (with the exception of (E)-2-hexenal) of *L. monocytogenes*, while only the exposure to carvacrol and thyme EO MIC values reduces the cultivability and significant cell load reductions were observed.

*Escherichia coli* was more sensitive to all the antimicrobials considered, not only in terms of cultivability but also in terms of membrane permeability and membrane potential. However, also for this strain, the antimicrobials used were unable to cause irreversible damages. In fact, the percentage of unstained cells of fragments remained constant and below the 3% independently on the strain, natural antimicrobial and the concentration used. As reported by Booyens and Thantsha (2014) during an antimicrobial treatment, the increase of the unstained population is related to a severe cell lysis or to a decreased staining accuracy due to formation of conglomerates. In addition, the increase of the unstained fraction is reported to be related with highly permeabilized or lysed cells unable to grow (Fröhling and Schlüter, 2015). The higher effect on membrane permeabilization of TO and PI staining compared to cF and PI dyes can be due to the fact that the effect of the last dyes was measured after a longer time span following the exposure to antimicrobials. It is presumable that the longer incubation after the stress exposure permitted the activation of microbial response mechanisms involving cell membrane repair. A wide literature shows that a precocious modification of cell membrane composition is pivotal for the microbial adaptation and survival in harsh conditions and sub-lethal stresses in order to maintain the proper fluidity, functionality, and permeability (Guerzoni et al., 2001; Gianotti et al., 2009; Mendoza, 2014; Serrazanetti et al., 2015; Siroli et al., 2015a). In fact, the immediate activation of the response mechanisms involving membrane modification was demonstrated both in fungi and bacteria to face even short time (lower that 100 min) physical-chemical stresses, including the exposure to natural antimicrobials (Guerzoni et al., 2001; Di Pasqua et al., 2006; Patrignani et al., 2008; Gianotti et al., 2009; Mendoza, 2014; Serrazanetti et al., 2015).

## Conclusion

The flow cytometry approach allowed to understand the targets of sub-lethal concentrations of citral, carvacrol, (E)-2-hexenal and thyme EO to *L. monocytogenes* and *E. coli* cells. The data showed that the membrane permeabilization is a common action mechanism of the antimicrobials considered on both strains. In contrast, they showed that esterase activity was not affected independent of strain, antimicrobial and its concentration. The approach used revealed that some antimicrobials such as citral, carvacrol and thyme EO were more effective against the Gram-negative strain used. These results are particularly important since Gram-negative bacteria are more resistant to many antimicrobials. However, the multiparameter data obtained showed that the natural antimicrobials and the concentrations used also caused reversible damages on *E. coli* MG 1655, the most sensitive strain tested, since the percentage of cell fragments remained constant even when the MIC values were used and when the membrane was depolarized. These data suggests that the levels used of citral, carvacrol, and thyme EO can be used as preservatives to control the growth of *L. monocytogenes* and *E. coli* only in combinations with other hurdles. In fact, concentrations capable of having lethal effects are incompatible with the food sensory features due to their low sensory threshold. Consequently, the detailed knowledge of the action mechanisms of natural antimicrobials considered in relation to the others hurdles applied is mandatory for their implementation at industrial level as preservation strategies. However, the implementation processes should be optimized in relation to the food matrices and production processes.

## Author Contributions

GB and FP contributed equally to the manuscript in the realization of lab trials. LS contributed to the statistical analysis. RL, OS, and AF contributed to the manuscript writing.

## Conflict of Interest Statement

The authors declare that the research was conducted in the absence of any commercial or financial relationships that could be construed as a potential conflict of interest. The handling Editor declared a past co-authorship with the authors OS and AF.
